# Seeing beyond the limit: A guide to choosing the right super-resolution microscopy technique

**DOI:** 10.1016/j.jbc.2021.100791

**Published:** 2021-05-18

**Authors:** Jessica Valli, Adrian Garcia-Burgos, Liam M. Rooney, Beatriz Vale de Melo e Oliveira, Rory R. Duncan, Colin Rickman

**Affiliations:** Edinburgh Super Resolution Imaging Consortium (ESRIC), Institute of Biological Chemistry, Biophysics and Bioengineering, Heriot-Watt University, Edinburgh, United Kingdom

**Keywords:** super resolution, microscopy, diffraction limit, fluorescence, localization, imaging, molecular imaging, molecular dynamics, protein–protein interactions, BiFC, bimolecular fluorescence complementation, DNA-PAINT, DNA-based point accumulation for imaging in nanoscale topography, dSTORM, direct stochastic optical reconstruction microscopy, ExM, expansion microscopy, FRAP, fluorescent recovery after photobleaching, NA, numerical aperture, PALM, photoactivated localization microscopy, PSF, point spread function, RESOLFT, REversible Saturable OpticaL Fluorescence Transition, SIM, structured illumination microscopy, smFRET, single-molecule FRET, SMLM, single-molecule localization microscopy, SOFI, super-resolution optical fluctuation imaging, SPT, single-particle tracking, STED, stimulated emission depletion, TIRF, total internal reflection fluorescence

## Abstract

Super-resolution microscopy has become an increasingly popular and robust tool across the life sciences to study minute cellular structures and processes. However, with the increasing number of available super-resolution techniques has come an increased complexity and burden of choice in planning imaging experiments. Choosing the right super-resolution technique to answer a given biological question is vital for understanding and interpreting biological relevance. This is an often-neglected and complex task that should take into account well-defined criteria (*e.g.*, sample type, structure size, imaging requirements). Trade-offs in different imaging capabilities are inevitable; thus, many researchers still find it challenging to select the most suitable technique that will best answer their biological question. This review aims to provide an overview and clarify the concepts underlying the most commonly available super-resolution techniques as well as guide researchers through all aspects that should be considered before opting for a given technique.

Optical microscopy has long been considered indispensable for biological research. Beginning with the development of cell theory in Hooke's iconic *Micrographia* in 1665 (1), microscopy techniques have underpinned many of the field's most exciting discoveries, from the theory of evolution (2) to observing molecular machines (3). Despite their successes, microscopy techniques have long been plagued by limitations imposed by the laws of physics, preventing these optical systems from resolving features below a certain size, known as the diffraction limit. The diffraction limit stems from a combination of the wave nature of light combined with the inability of our optical systems to focus these waves to a single point below a certain diameter. This limit, which determines the minimum distance that needs to separate two points in order for them to be resolved by a given optical system, was first defined in 1873 by the German physicist, Ernst Abbe (4, 5). This distance is dependent on the wavelength of light, refractive index of the medium through which that light travels, and angles of diffracted light that can be collected by the microscope objective. The latter two of these factors are used to calculate the numerical aperture (NA) of specific objectives, which represents a unitless measure of the range of angles across which that objective can accept incoming light, and thus affects the resolution achievable with that objective. Indeed, Abbe (4, 5) showed that the diffraction limit is roughly equal to the wavelength of light (λ) divided by twice the NA for lateral resolution, and to roughly 2λ/NA^2^ for axial resolution. This means that for a standard fluorescence system, such as a confocal microscope operating in the visible spectrum, the resolution is limited to around 170 to 250 nm laterally and around 470 to 670 nm axially, when detecting wavelengths between 470 and 700 nm.

Prior to 2000, the only techniques able to image subdiffraction features were near-field-based techniques, which use nanometric detectors placed very close to the sample to detect evanescent waves (6). Since the turn of the millennium, however, an increasing number of far-field techniques have emerged that are able to overcome the diffraction limit, and these will be the focus of this review. Super-resolution techniques can be broadly split into two categories: super-resolved ensemble microscopy techniques, which improve the resolution of overall structures, and super-resolved single fluorophore microscopy techniques, which use localizations of individual fluorescent molecules to build up an overall structure. REversible Saturable OpticaL Fluorescence Transition (RESOLFT) ensemble techniques include stimulated emission depletion (STED) microscopy and the similar ground state depletion/RESOLFT techniques, structured illumination microscopy (SIM), pixel reassignment techniques, and arguably the sample preparation–based expansion microscopy (ExM). Single-fluorophore techniques, meanwhile, include the collective single-molecule localization microscopy (SMLM) techniques, most notably direct stochastic optical reconstruction microscopy (dSTORM), photoactivated localization microscopy (PALM), and DNA-based point accumulation for imaging in nanoscale topography (DNA-PAINT).

Since their inception, super-resolution techniques have steadily gained in popularity, with these techniques leading to ever increasing discoveries that previously eluded detection. Indeed, the impact of super-resolution techniques on research has been such that the 2014 Nobel Prize in Chemistry was awarded to Stefan W. Hell, Eric Betzig, and William E. Moerner; Stefan Hell for the development of STED microscopy, and Eric Betzig and William Moerner for work leading to the development of PALM and subsequent single-molecule localization techniques (7). Given the wealth of available super-resolution techniques, it can be a nontrivial task to select that which will provide the right information to address a specific research question. This review therefore aims to provide insight into the important considerations for selecting the best super-resolution technique for the problem at hand. This review does not focus on an in-depth explanation of each super-resolution technique but rather seeks to help biologists assess which capabilities are most important for answering their research question and to thus select the most compatible super-resolution technique for their needs.

## Brief overview of key super-resolution techniques

### Ensemble super-resolution techniques

**Figure 1:**
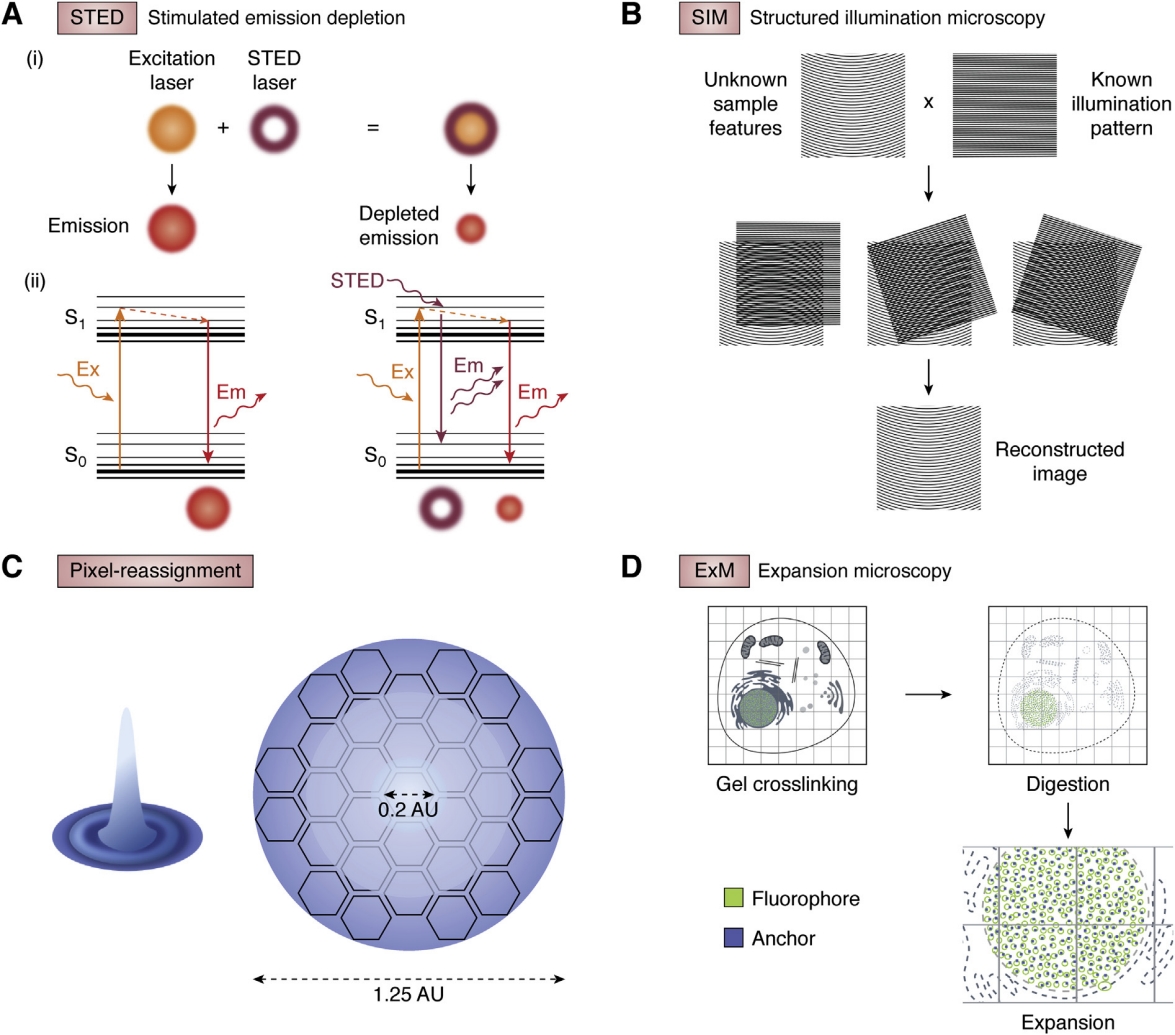
**Ensemble super-resolution microscopy techniques.***A*, stimulated emission depletion (STED) microscopy: (i) STED laser is aligned with excitation laser and depletes emission of fluorescent molecules in overlapping regions; (ii) depletion occurs though interruption of internal conversion process undergone by excited electrons, forcing excited fluorescent molecules to immediately return to ground state, resulting in the release of a photon with a shorter lifetime and a red-shifted wavelength that can be excluded from detection by band filtering. *B*, structured illumination microscopy (SIM): patterned light interferes with high-frequency sample details to produce lower-frequency Moiré fringes used to reconstruct a super-resolved image. *C*, pixel reassignment example—Airyscan detector: confocal pinhole substituted with 32-element detector array; each detector is equivalent to a 0.2 AU pinhole, but the array maintains light collection efficiency of a 1.25 AU pinhole. *D*, expansion microscopy: samples are embedded within a swellable hydrogel; fluorophores are anchored to the gel before crosslinking and digestion of cellular structures and finally expansion.

STED is a confocal laser scanning–based technique, with super-resolution achieved through the addition of high-powered torus-shaped STED lasers. The STED lasers are aligned with the excitation beam and deplete the emission of fluorescent molecules in the overlapping region (Fig. 1*A*i) (8, 9). This depletion is achieved through interruption of the internal conversion process undergone by excited electrons, with the high-energy depletion laser forcing the excited fluorescent molecules to immediately return to the ground state. This results in the release of a photon with a wavelength equal to the depletion laser wavelength used, which is easily removed with filters (Fig. 1*A*ii) (8, 9). STED was initially implemented using two-photon lasers for depletion but is now commonly commercially implemented with continuous wave (CW) lasers or pulsed lasers with longer pulse lengths (8, 9). CW depletion lasers simplify implementation by removing the need for synchronization of laser pulses (10); however, this results in the sample being exposed to the STED beam between excitation pulses, when it does not contribute to image formation, increasing photobleaching. Unlike pulsed STED, where all the depleting photons arrive shortly after excitation of the sample, CW implementations suffer from the added issue of lower instantaneous STED intensities meaning that a greater number of molecules are not exposed to enough STED photons to be depleted, degrading the attainable resolution (11). This has given rise to time-gated STED, in which short-lifetime emitted photons are removed by time gating as they are unlikely to have had sufficient opportunity to undergo STED (11, 12). Pulsed implementations also benefit from time gating. Commercial pulsed systems use STED lasers with a ~1 ns pulse length, as compared with the picosecond or femtosecond 2-photon lasers initially used. The longer pulse length allows for the laser power to spread over a longer duration, reducing photobleaching (13), and can also overcome issues observed with shorter pulse lengths including polarization effects (14) and laser synchronization jitter (15). STED efficiency is slightly lower with longer pulse lengths, but this reduction is eliminated through the use of time gating (16).

Details within samples are detected by microscopes as combinations of light waves of different frequencies. However, microscope optics are inherently limited in which frequencies they can sample, meaning the highest-frequency information (*i.e.*, the highest-resolution details in the sample) are lost. SIM techniques take advantage of a phenomenon known as the Moiré effect, whereby the interference of two differing high-frequency patterns when overlaid will produce a sum of the frequencies (very high) and the difference between the frequencies (low). Therefore, by using an illumination pattern just below the diffraction limit of the microscope, it is possible to move high-frequency information in the sample into the sampling range of the microscope. By controlling the orientation and phase of the illumination pattern, the original high-frequency information can be back-calculated from the detected image (Fig. 1*B*) (17). To achieve a full reconstruction, several images need to be collected with the illumination patterns in different positions and orientations to reconstruct the final SIM image (Fig. 1*B*) (17).

Laser scanning confocal-based systems generally acquire data point by point onto a detector comprising a single pixel. Pixel reassignment techniques improve spatial resolution by combining point-scanning illumination with multipixel array detectors to create a higher spatial frequency component of an image. The central pixel of the adapted detector array plays the role of the confocal pinhole, but the additional pixels allow more light to be collected and provide additional structural data acquisition. Perhaps the best known of these techniques is Zeiss's Airyscan, which uses a detector composed of 32 elements, each equivalent to a point detector with a 0.2 airy unit pinhole (Fig. 1*C*) (18). Each element of the detector array acts as a separate pinhole, meaning that the fluorescence is captured 32 times rather than once. By correcting for the displacement of each element from the optical axis, the image can be reconstructed with a higher spatial resolution (18).

ExM relies not on adapting the microscope to surpass the resolution limit but rather on physical expansion of the sample itself, which increases the effective resolving power of a diffraction-limited microscope. This is achieved by embedding of the sample within a hyperswellable hydrogel with long chains and cross-linking agents, creating an extremely dense linked mesh (19). During gelation, proteins and fluorophores are anchored to the gel. The permeated sample is then mechanically homogenized with various techniques (high temperature, detergents, proteases, etc) to avoid resistance from the sample to the expansion, before addition of water that triggers swelling of the polymer, up to 100× its original size (Fig. 1*D*) (19). Expanded samples can then be imaged on a standard widefield or confocal microscope.

### Single fluorophore detection techniques

**Figure 2:**
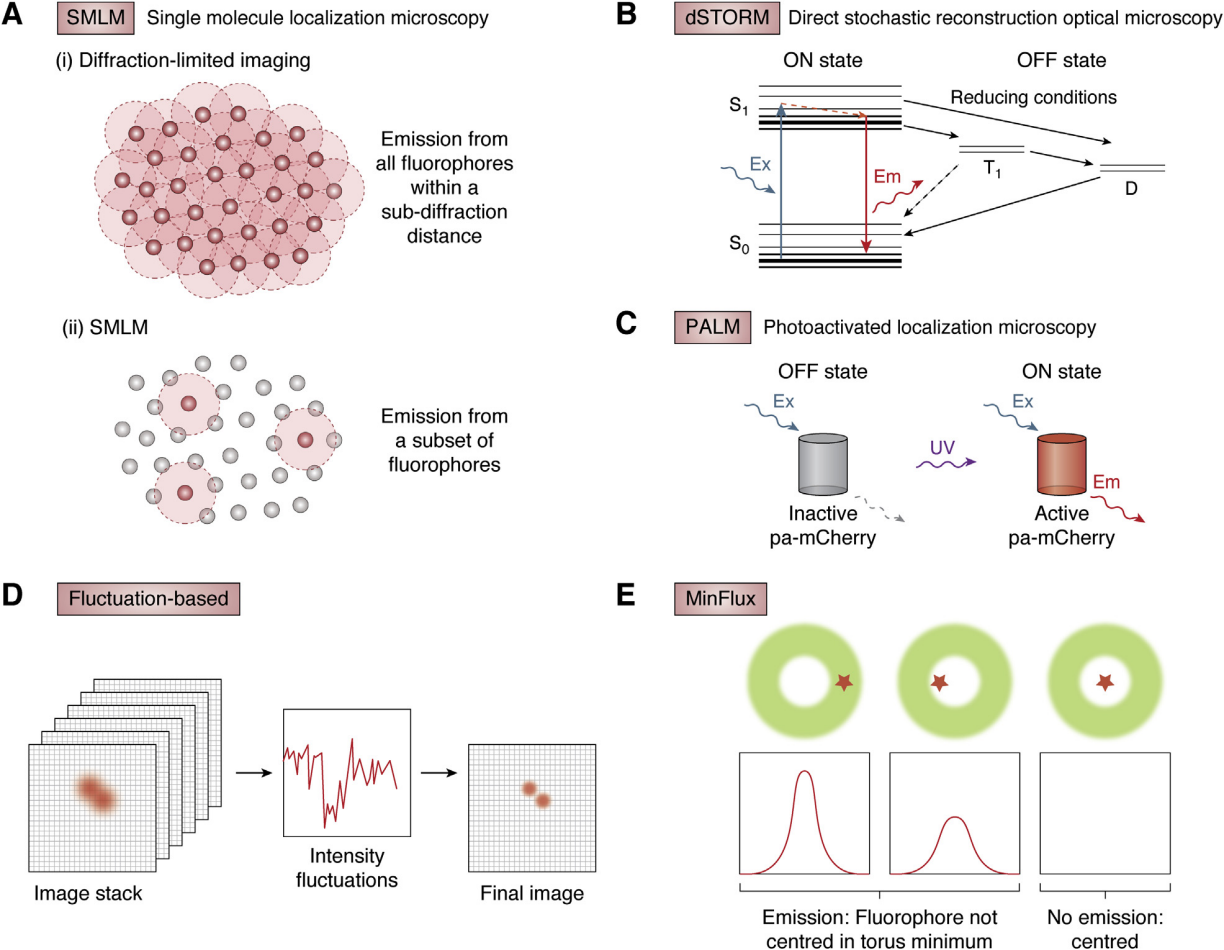
**Single fluorophore detection–based super-resolution microscopy techniques.***A*, principle of SMLM. *B*, dSTORM uses buffers with reducing conditions to drive fluorescent molecules into short-lived (T1) or long-lived (D) dark states to cause molecules to blink. *C*, PALM uses photoactivatable fluorophores that need to be activated by 405 nm light before being excited. *D*, fluctuation-based example—SOFI: intensity fluctuations from emitters tracked through time-lapse images, then used to refine emitter localization. *E*, MinFlux: blinking fluorophores are combined with a torus-shaped excitation beam with a central intensity minimum; determining the point with minimum emission provides precise localization coordinates. dSTORM, direct stochastic optical reconstruction microscopy; PALM, photoactivated localization microscopy; SMLM, single-molecule localization microscopy; SOFI, super-resolution optical fluctuation imaging.

SMLM techniques are numerous and varied, but all rely on the same principle of being able to differentiate individual fluorophores in time rather than in space; fluorophores within subdiffraction distances, which cannot be differentiated when fluorescing at the same time (Fig. 2*A*i) can be pinpointed if they fluoresce one by one (Fig. 2*A*ii). Individually fluorescing fluorophores will emit hundreds to thousands of photons, producing a diffraction-limited intensity pattern, and when multiple fluorophores are within subdiffraction distance of one another, their emission patterns will overlap (20, 21). In the absence of emission from neighboring fluorophores, the light from a single fluorescing fluorophore will still be diffraction limited, but the distribution of the photons it emits can allow for calculation of a central localization with subpixel precision (20, 21). All SMLM techniques therefore rely on having dyes or fluorophores switch between on and off states, but each technique achieves this in a unique way.

The most commonly used SMLM techniques are dSTORM and PALM. dSTORM uses specialized buffers to drive standard organic fluorescent molecules into long-lived dark states, in which the fluorophores cannot be excited without first returning to the ground state (Fig. 2*B*) (22, 23). The optimal buffer composition is selected to maximize the rate of entry into the dark state and minimize rates of escape from this state or photobleaching. This is commonly achieved through the inclusion of reducing buffers and reduction of dissolved oxygen. If a sufficiently large proportion of fluorophores reside in this long-lived dark state at any one time, molecules returning to the ground state will momentarily emit photons and their detected emissions will be isolated. A number of publications have focused on optimal buffer composition and can provide further information on the subject (24, 25, 26). PALM, on the other hand, uses specific photoactivatable or photoswitchable fluorophores, which are generally genetically encoded. The most common photoactivatable fluorophores are natively found in an off state, in which illumination with the excitation wavelength will not produce fluorescence (27). Illumination of the fluorophores with an activation wavelength, often UV, causes a conformational change that then allows the fluorophore to be excited by the excitation wavelength to produce fluorescence (Fig. 2*C*) (27). Photoswitchable fluorophores, in comparison, normally photoconvert between a shorter fluorescent species to a longer wavelength form, and this can be one way or reversible (28). Detection of single-molecule emission in this technique is therefore achieved by cycling low levels of activation light to turn on or switch a subset of the fluorophores and then allowing these to bleach (27). Although organic dyes are typically brighter and more photostable than fluorescent proteins, there is extreme variability between different dyes, and these properties also depends upon experimental conditions (29). Choice of dye or fluorophore is therefore important as the number of photons will directly affect localization accuracy (20).

Fluctuation-based techniques such as Super-resolution Optical Fluctuation Imaging (SOFI) (30) and Super-Resolution Radial Fluctuations (31) take advantage of natural variations in the intensity of light emitted by fluorophores as they transition through fluorescent and nonfluorescent states (Fig. 2*D*). Unlike SMLM techniques, fluctuation-based super resolution does not detect or localize individual fluorophores but instead calculates statistical properties of the variations across the entire frame (30, 31). The more probes fluctuate, the better the achievable resolution will be, meaning these techniques therefore work very well with dSTORM or PALM samples; however, they are also compatible with standard fluorescent samples prepared for widefield or confocal imaging.

Minimal photon flux imaging (MINFLUX) combines the concepts of STED and SMLM to achieve nanometer resolution. For MINFLUX imaging, fluorophores are required to blink or switch as in dSTORM or PALM, but this is combined with excitation using a torus-shaped beam with an intensity minimum at the center (32). This means that fluorophores exactly under the centre of the beam will not be excited, and this can be used to pinpoint precise coordinates for their localization (32). Excitation at multiple positions is used to centre in on the excitation minimum as emission intensity will decrease as the fluorophore position approaches this minimum (Fig. 2*E*) (32). In contrast with many other techniques, this approach is very photon efficient, requiring up to 100-fold fewer photons to achieve equivalent resolutions to state-of-the-art SMLM techniques (33). Moreover, MINFLUX far surpasses these techniques in both lateral and axial localization precision (34). However, MINFLUX is a relatively computationally intensive technique and, having first been commercially produced in December 2019, is not widely available.

The different available super-resolution techniques each encompass distinct strengths and limitations. When designing a super-resolution experiment, it is thus vital to select a technique that can appropriately answer the core biological question. Biological questions that can be investigated using super-resolution microscopy can be broadly categorized into those concerned with defining high-resolution structures of interest, those concerned with live-cell dynamics, and those concerned with molecular interactions. The following sections will discuss each of these categories and the important factors in selecting a super-resolution technique within each.

## Selecting a super-resolution technique for defining structures of interest

Perhaps the most obvious and frequent application of super-resolution microscopy is in addressing questions of subcellular localization or colocalization, which require precision beyond the diffraction limit. While most super-resolution techniques will allow for a labeled target to be localized at subdiffraction resolution, improvements in spatial resolution will always be met with a trade-off in other abilities of the system, such as temporal resolution, multicolor imaging, or avoidance of phototoxicity/photobleaching.

**Figure 3:**
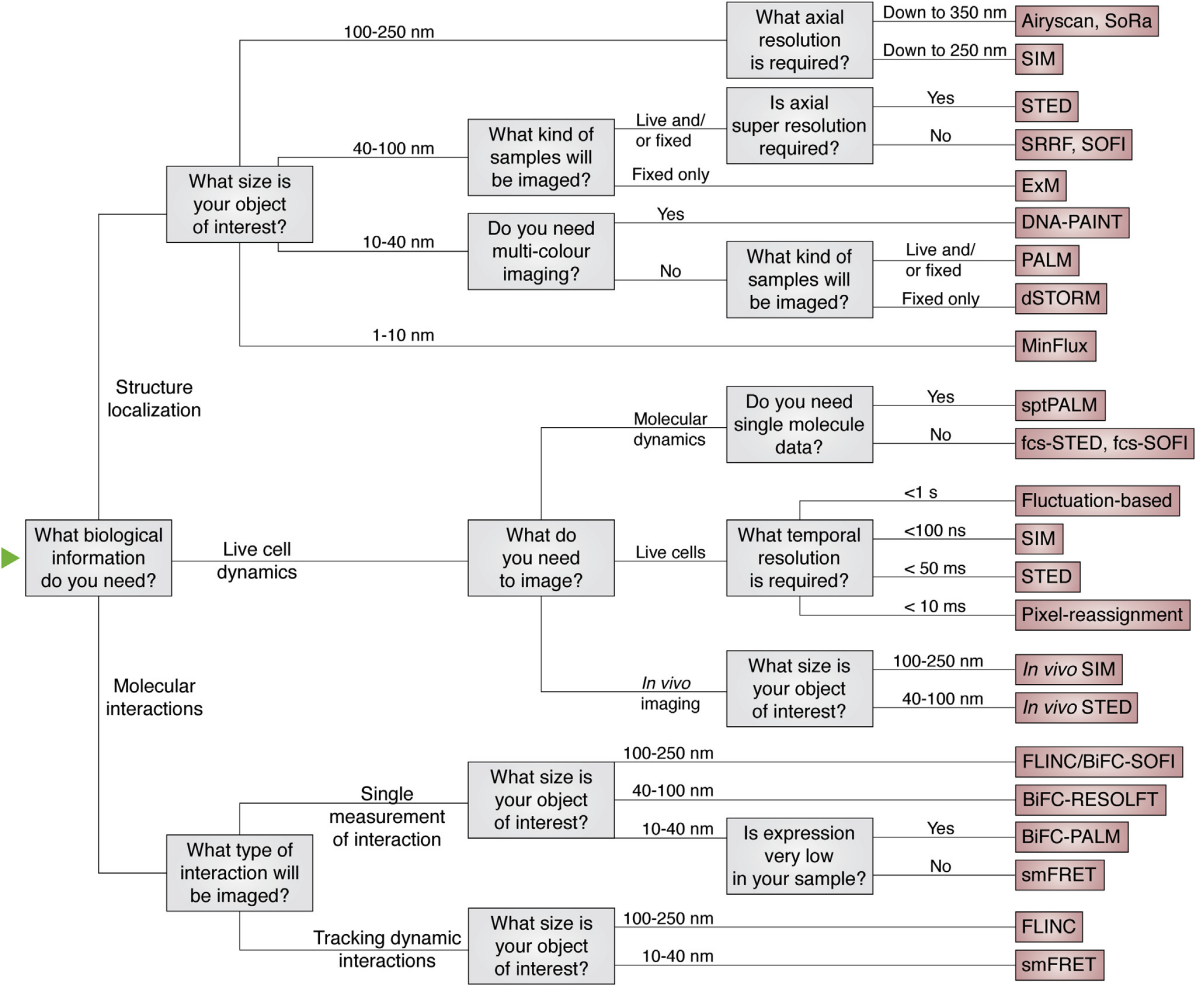
**Simplified guide to choosing a super-resolution technique**.

It is therefore essential to consider the requirements of each specific experiment to determine which super-resolution technique will provide optimal image data. While detailed considerations are discussed later, a simplified flow diagram for selecting the appropriate super-resolution technique is presented in Figure 3.

### Resolution

**Figure 4:**
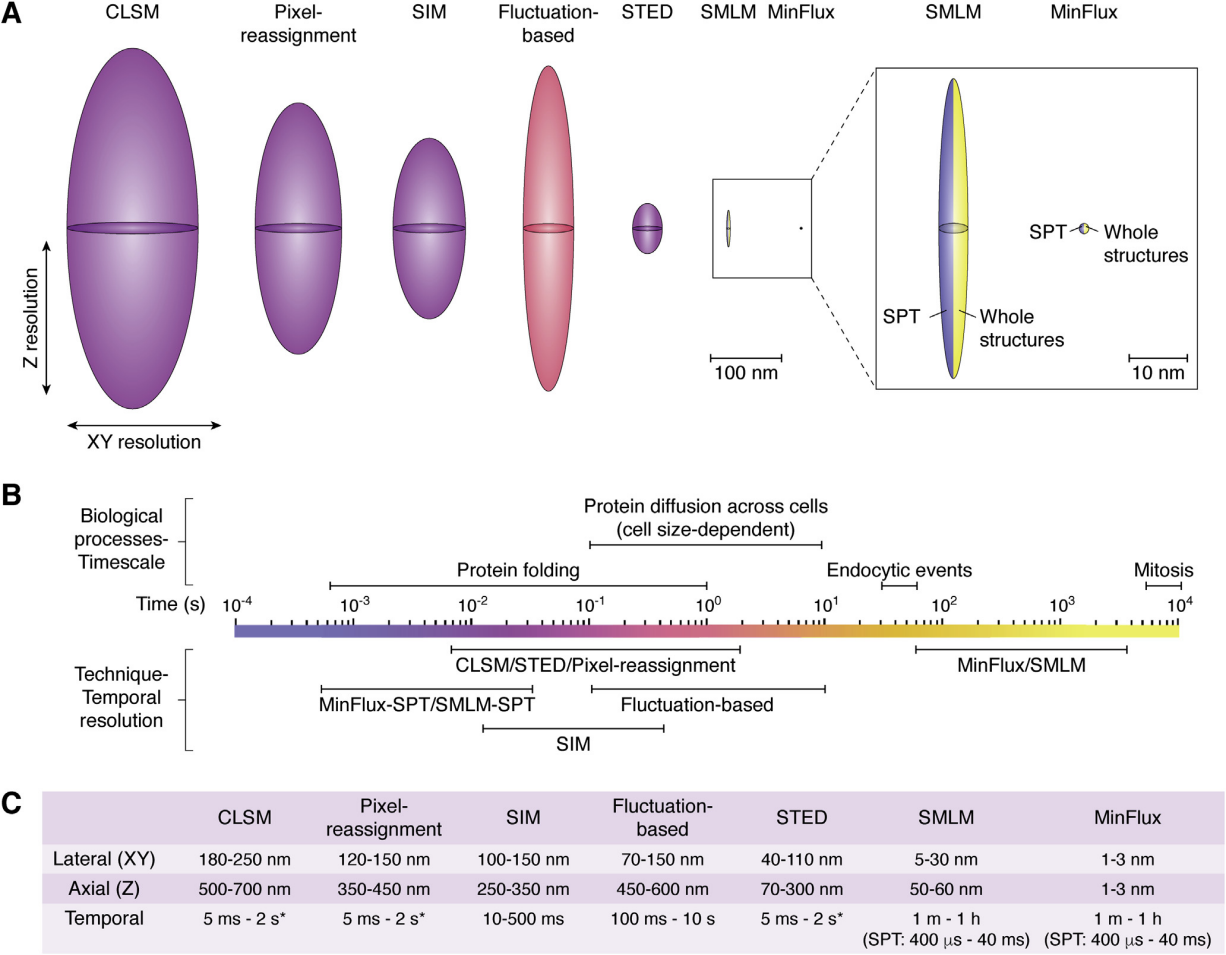
**Spatiotemporal resolutions achieved by different super-resolution techniques as compared with confocal laser scanning microscopy (CLSM).***A*, point spread functions (PSFs) for each technique at minimal lateral (XY) and axial (Z) resolutions typically achievable on biological samples. Color coded according to temporal resolution scale in *C*. Adapted from Ref. (124). *B*, temporal resolution of each technique compared with timescales of biological processes. For single fluorophore detection techniques, such as SMLM and MINFLUX, each individual localization and the tracking of single particles can be acquired very rapidly, while imaging of gross structural changes requires numerous localizations and is relatively slow. *C*, ranges of lateral, axial, and temporal resolutions typically achieved using each technique on biological samples. ∗Temporal resolution is highly dependent on imaging area for laser scanning techniques. SMLM, single-molecule localization microscopy.

The first aspect that should be taken into consideration is the resolution required to answer the biological question. The resolution of an optical system can be defined as the smallest distance between two points at which the two can still be distinguished as separate entities. The resolution of super-resolution techniques varies widely (Fig. 4)—from pixel reassignment techniques, which produce around a 1.4× improvement in resolution or an effective resolution of down to 120 nm (35, 36), to the recently developed MINFLUX technique, which has a reported resolution of down to 1 nm (34). Techniques such as STED and SIM inherently allow for improvements in axial resolution, albeit to different extents; however, SMLM applications will generally require adaptations such as deformation of the point spread function (PSF) to provide axial super resolution (37).

The differences in resolution of these techniques make them suited to different questions of localization. For example, detailed analysis of mitochondrial morphology is often performed using less high-resolution techniques, such as Airyscan (38, 39). Localization of proteins to specific membrane domains, on the other hand, can involve resolving distances of under 50 nm and are therefore better suited to higher resolution techniques. For example, SMLM techniques have been widely used for localization of receptors within membranes (40, 41, 42, 43), and MINFLUX has recently been used to analyze the organization of mitochondrial contact site and cristae organizing system components, suggesting that several molecules of the Mic60 protein form rings at individual crista junctions (44).

While it might be tempting to always opt for the highest-resolution system available, this may in fact detract from the usable information encoded within the image data by sacrificing other necessary information. When selecting a super-resolution technique, one should therefore consider all the factors inherent in the experimental setup, which will affect the data acquired.

### Multicolor localization

Another major consideration will be the suitability of different techniques to multicolor imaging. While SIM, fluctuation-based and pixel-reassignment techniques, and ExM have no specific requirements in terms of fluorophore selection and are therefore easily compatible with multicolor imaging, this is not the case for all super-resolution techniques. Multicolor STED is possible, with up to four-color imaging having been demonstrated to date (45). However, this is dependent on the STED lasers present on the system, and the availability of efficiently depleted fluorophores can become limited as the number of desired colors increases (46).

Multicolor imaging can be particularly difficult when it comes to single-molecule localization. PALM uses photoactivatable or photoswitchable fluorophores, and thus far, most currently available options either emit green or red fluorescence upon activation or switch from green to red. This means that options for multicolor imaging by PALM are generally limited to two-color experiments (47, 48). Similar limitations exist for dSTORM, as many dyes with good blinking properties have different buffer requirements for inducing blinking, making it difficult, though not impossible, to combine these for multicolor imaging (49, 50, 51). PALM and dSTORM can also be combined to increase multicolor options (52). In all cases of multicolor SMLM, it is important to consider balancing the brightness of different channels. Multicolor imaging can either involve acquisition of channels simultaneously or sequentially. With sequential acquisition, illumination and detection sensitivity can be optimized for each fluorophore/dye, but this will increase overall imaging time and require drift correction between the channels (53). Simultaneous acquisition, meanwhile, can be performed on a single camera with the detector split between the two channels, though this does not allow for independent adjustment of detection sensitivity, or on two cameras (54). Both these simultaneous options, however, require registration of the two channels in postprocessing, which needs to be performed at super-resolution levels of correction, which is nontrivial (55). Finally, multicolor SMLM suffers from issues of chromatic aberration, resulting in different resolutions and lack of colocalization of different wavelength fluorophores (56).

DNA-PAINT represents an alternative option for single-molecule localization, which has more opportunities for multicolor imaging. In DNA-PAINT, molecules of interest in the sample are labeled with antibodies bound to single DNA strands (57). Complementary DNA strands labeled with fluorophores are introduced into the solution, and these transiently bind to the complementary strands on the sample (57). Blinking in DNA-PAINT does not require special buffers or photoswitching/photoactivation but instead results from dye molecules being immobilized in the imaging plane during the transient binding events (57). Multicolor imaging through DNA-PAINT is therefore straightforward as different dyes can be coupled to target-specific DNA sequences. Multicolor imaging can be taken even further through the use of exchange-PAINT, whereby sequential use of DNA-PAINT labels with washing in between allows for essentially unlimited use of even a single color fluorophore for labeling different structures of interest (57).

### Sample thickness

The choice of technique for simple localization experiments will also be impacted by the thickness of the sample and depth of the region to be imaged within that sample. Thicker samples suffer from increased out-of-focus light, and imaging deep within samples increase spherical aberration because of refractive index mismatch between the sample and the optical elements (58). Both these factors will influence lateral and axial resolution, though certain techniques are able to minimize these effects. SMLM techniques are commonly performed on total internal reflection fluorescence (TIRF) setups, limiting imaging to structures very close to the coverslip. TIRF microscopes use a laser inclined past a critical angle (typically between 50° and 70°) and a mismatch in refractive index of the immersion medium and the sample medium to cause the laser light to reflect off the coverslip (59). This results in illumination of the sample by an evanescent field emanating from the laser light (59). Penetration of this evanescent wave is dependent on excitation wavelength and the NA of the objective but typically range from under 100 nm to a maximal penetration of around 200 nm (60). While this is ideal for studies of membrane biology, including secretion and exocytosis (61, 62), it precludes imaging of entire cell volumes or of organelles deeper within the cell. SMLM techniques are also compatible with highly inclined laminated optical sheet illumination, where the laser passes through the sample at a sharp angle, allowing imaging of a diagonal slice up to a depth of 10 μm, and can be easily performed on TIRF setups but only allows for a small field of view (63). Even thicker samples can be imaged through the use of selective plane illumination microscopy (64). For monolayer samples, 3D SMLM is also achievable in widefield setups, for example, through engineering of the PSF to produce astigmatism whereby a cylindrical lens breaks the symmetry of the PSF above and below the focal plane (65) or by using two focal planes as in biplane imaging (58).

Standard SIM imaging is based on a widefield setup and is therefore sensitive to out-of-focus light, making it difficult on samples thicker than 5 to 10 μm (66). However, adapted SIM systems with slit-confocal structured illuminations have shown success in blocking out-of-focus light allowing for SIM imaging of thicker samples (67). Confocal-based techniques, such as STED, naturally provide optical sectioning, removing out-of-focus light. However, these techniques remain susceptible to spherical aberration at greater imaging depths (68). Thicker samples will also suffer from issues of light penetration regardless of the super-resolution technique; this can be overcome by combining super resolution with two-photon microscopy. Two-photon excitation has been successfully combined with multiple super-resolution techniques, including PALM (69), STED (70, 71), and SIM (72, 73).

ExM can provide an alternative solution for mitigating penetration issues, as the expanded sample inherently undergoes decrowding of biomolecules, and the final sample is composed of 99% water, making it relatively optically transparent (19). It is, however, worth noting that this expansion will also result in a much larger imaging volume, resulting in much longer imaging times for large or 3D acquisitions.

### Other considerations

Choice of super-resolution techniques may also be affected by the need for quantitative analysis. This is particularly relevant in the use of SMLM for analyzing clustering of proteins. In theory, single-molecule imaging should allow for absolute determination of numbers of molecules within cellular structures. In practice, however, there are numerous factors that can contribute to artifacts such as overcounting and artificial clustering.

Approaches that include labeling with antibodies can be particularly problematic as multiple antibodies may bind a single protein of interest producing multiple localizations per labeled molecule (74). Antibodies can also induce artificial clustering by crosslinking proteins to one another (75). For true measures of protein stoichiometry and clustering, it is far better to use one-to-one tagging of proteins either *via* genetically expressed fluorophores or through the use of self-labeling enzymes. Moreover, the size of labels on structures of interest should be considered. Typical IgG antibodies are generally around 10 to 15 nm in size; labeling of structures with primary and secondary antibodies could therefore result in a signal 30 nm away from actual protein of interest, which is particularly problematic as the resolution of the imaging technique improves (76). This linkage error can be minimized through the use of smaller labeling molecules, for example, primary antibody antigen-binding fragments bound directly to fluorescent dyes, or through non-IgG–based techniques, such as labeling with nanobodies or aptamers (76, 77, 78).

Additional complexity arises from the blinking of dyes, which produces multiple localizations per dye or fluorophore (74). PALM should theoretically overcome this issue, with each fluorophore being activated and continuously imaged until photobleached, and therefore only counted once. However, this model is somewhat oversimplified as there is evidence that photoactivatable fluorophores are also able to enter dark states during PALM imaging, resulting in blinking and multiple localization counts (79). While complex modeling of blinking properties can be used to somewhat address these issues during analysis, these blinking kinetics vary between dyes/fluorophores and can be affected by environmental conditions and photobleaching (29, 80). The development of qPAINT has sought to overcome these issues by basing quantitation on predictable label-binding kinetics rather than complex dye kinetics and has the added advantage of being immune to photobleaching as the transient binding means that fresh labels are constantly replacing bleached molecules (81). It is beyond the scope of this review to cover the very important aspects of data processing strategies, avoidance of artifacts, and quantifying SMLM data. This has been covered extensively elsewhere (56, 82, 83).

Finally, it is worth noting that while some super-resolution techniques (*e.g.*, super-resolution radial fluctuation, ExM, SMLM) can be performed on standard widefield or confocal systems, others will require expensive equipment and may not be as ubiquitously accessible within research departments. However, specialized super-resolution facilities are becoming more widespread, including open access facilities such as our own, catering to a larger research community.

## Selecting a super-resolution technique for assessing live-cell dynamics

Not all imaging experiments will focus simply on static questions of localization but may in addition incorporate questions of live-cell dynamics. While the considerations discussed previously remain relevant, additional factors come into play for super-resolution imaging of live samples, tracking of molecular dynamics, and even *in vivo* imaging in live animals, and these are discussed below.

### Live-cell imaging

When it comes to live-cell imaging, it is not only lateral and axial resolution that are important but also temporal resolution. This represents the speed at which sequential images can be captured and will define what type of processes can be analyzed. Cellular dynamics can occur on a time scale of microseconds to days and will thus be suited to different imaging techniques. Several super-resolution techniques require acquisition of multiple raw frames to produce an image of global structures in the sample; typically 9 to 15 for 2D or 3D SIM, hundreds for fluctuation-based techniques, and thousands for SMLM (84). This causes obvious limitations in the achievable frame rate for tracking live-cell dynamics (Fig. 4*B*).

Other considerations for live-cell super resolution include phototoxicity and suitability of labeling strategy. While photodamage and photobleaching will also affect fixed cell experiments, phototoxicity may be one of the primary considerations for live-cell imaging, as it may disrupt the very processes being analyzed. Two main factors that will affect degree of phototoxicity are illumination wavelength and illumination intensity. There is clear evidence that irradiation sensitivity worsens with decreasing excitation wavelength (85, 86). For certain techniques, excitation with particularly phototoxic 405 nm light is unavoidable; many photoactivatable or photoswitchable fluorophores used for PALM, for example, are activated by 405 nm illumination (87). In these cases, photodamage can be reduced by minimizing total irradiation time to reduce the photon burden on the sample (86). Conversely, other techniques such as STED are compatible with longer wavelength dyes and fluorophores but require the use of high-powered depletion lasers with irradiation intensities 3 to 5 orders of magnitude higher than the excitation intensities (86). While phototoxicity does not necessarily make these techniques incompatible with live-cell imaging, stringent tests should be employed to ensure that irradiation does not impact the cellular processes being imaged (88). The gentlest techniques for live-cell imaging are generally pixel reassignment techniques or SIM, which require much lower irradiation intensities, and these may therefore be the most appropriate for long time-lapse imaging experiments.

Labeling strategy is particularly important for live-cell dSTORM experiments. While single-molecule imaging in live cells is generally performed by PALM, live-cell dSTORM is not impossible. However, the buffers with reducing and oxygen-scavenging properties that are generally used to induce blinking of dyes will cause stress to live cells and should be avoided (86). The intracellular environment does itself display these properties, albeit to a lesser extent, meaning that live-cell dSTORM is possible without the use of stress-inducing buffers with the use of selected dyes such as TMR-Star or Alexa Fluor 647, which will blink even under these milder conditions (89, 90).

A number of super-resolution techniques have even been applied to *in vivo* imaging in live animals. However, these techniques generally require adaptation to overcome the complexities of *in vivo* imaging, including optical aberrations introduced by deeper imaging and vibrations caused by vital functions such as heart beats, which can introduce image distortion. *In vivo* SIM has been used to image live zebrafish larvae and mice brains, overcoming the aforementioned issues using adaptive optics in combination with shorter frame acquisition times and frame-to-frame registration (91). *In vivo* STED, meanwhile, has been successfully implemented to super resolve neurons and synaptic proteins in living mouse brains with the use of a custom advanced mounting stage to reduce thermal drift and sample-induced vibrations (92, 93).

### Molecular dynamics

Traditional techniques for determining molecular dynamics have historically consisted of fluorescent recovery after photobleaching (FRAP) or fluorescence correlation spectroscopy, both of which measure diffusion of fluorescent molecules. While both techniques can provide useful information on real-time dynamics, they remain diffraction limited and can only provide average rather than molecule-specific data. Both these have been combined with super-resolution techniques. Fluorescence correlation spectroscopy, for example, has been combined with SOFI to correlate diffusion dynamics with subdiffraction mapping of pores within materials such as hydrogels (94, 95), and with STED to assess lipid membrane dynamics (96, 97). FRAP, meanwhile, has been combined with SMLM to assess membrane dynamics of transforming growth factor-β receptors, showing that this receptor is transported to the plasma membrane primarily in monomeric form (98). In this approach, FRAP was used to bleach existing fluorescent molecules at the basal cell membrane, and SMLM was then performed to get super-resolved information on recruitment of new receptor molecules (98).

Analysis of molecular dynamics on an individual molecule basis can also be performed through single-particle tracking (SPT) techniques. Not all single-molecule techniques are compatible with SPT in cells; as discussed previously, buffers for dSTORM are incompatible with live samples, and DNA-PAINT generally requires antibody labeling of fixed samples. There are, however, options for SPT using either sptPALM or adapted PAINT techniques, such as Tag-PAINT (99), which binds DNA docking strands to SNAP or Halo Tags, and LIVE-PAINT (100), which uses genetically expressed peptide sequences rather than antibody-linked DNA labels.

## Selecting a super-resolution technique for assessing molecular interactions

A final experiment type that will have additional considerations to those discussed previously relates to questions of protein–protein interactions or interactions of other molecules. While molecular interaction techniques have historically been able to provide information about interactions occurring at <10 nm distances, they could not spatially resolve these interactions across subdiffraction features within a sample. The advent of super-resolution microscopy has now allowed for subdiffraction regional differences in these interactions to be observable, providing new insight as compared with historical techniques, which provided average information from across a sample. Selection of a technique for these types of experiments is less clear cut, and for the best evidence of molecular interaction, these techniques should be combined with biochemical assays to show more than just proximity. However, super-resolved microscopy techniques can still be invaluable in these types of experiments as they can combine interaction data with subdiffraction location information.

Microscopy techniques can be used to detect interprotein interactions and even intraprotein interactions in terms of conformational changes. The standard microscopy technique for assessing these interactions is FRET (101). This technique measures the process of transfer of energy from a donor fluorophore to an acceptor fluorophore when these come into close proximity (102). Standard FRET techniques do not provide a spatially super-resolved image but do provide information about interactions occurring at 1 to 10 nm distances (102). However, FRET has also been combined with super-resolution techniques to provide single-molecule FRET (smFRET). smFRET was first implemented in 1996 using near-field microscopy techniques that overcome the diffraction limit by minimizing the distance between the detector and sample (<10 nm) (103). Newer applications of smFRET use quantum dot–based FRET nanosensors (104, 105) or SMLM-like blinking methods such as photoactivatable (106) or PAINT-based FRET probes (107, 108) to enable single molecule–level detection. While smFRET is usually performed *in vitro* on free proteins in solution, it has been demonstrated within live cells to show processes such as individual SNARE proteins interacting with membrane-tethered complexes (109) and conformational changes of rapidly accelerated fibrosarcoma proteins (110). smFRET is more widely applicable to analyzing other molecular interactions, such as protein–DNA interactions (111, 112), DNA–DNA interactions (113, 114), and protein–RNA interactions (115, 116), to name but a few.

Protein–protein interactions can also be assessed at high resolution and single-molecule level through combinations of super-resolution techniques with bimolecular fluorescence complementation (BiFC). BiFC involves fusing proteins of interest to two nonfluorescent fragments of a fluorophore, which will interact to produce fluorescence when in close proximity (117). BiFC has been successfully combined with SOFI (118), RESOLFT (119), and PALM (120, 121). Finally, fluctuation-based super-resolution techniques such as SOFI can be combined with fluorescence fluctuation increase by contact, whereby fluctuations of fluorescent proteins will speed up when they come into close proximity (122). The super-resolution technique with which interaction detection methods are combined will determine the spatial resolution that is achievable. BiFC can provide a more sensitive measure of protein–protein interactions, as even small fractions of complementary fluorophore fragments will combine and fluoresce (123). However, this generation of fluorescence is irreversible and cannot be used to track dynamics (123). smFRET and fluorescence fluctuation increase by contact are both reversible processes and are thus more powerful in determining spatiotemporal dynamics (123).

## Conclusions

Anticipating what an experiment should achieve at the outset is key to any successful imaging experiment. Careful consideration of the available methods is therefore vital to ensure that the correct technique is used to fully answer the scientific question. There is a lot to take into account: the specimen itself (size, live/fixed, thickness, opacity, etc), the aspect of the specimen under study (*i.e.*, cell structures, surfaces, single molecules), and the temporal sensitivity required (*i.e.*, for molecular dynamics, interactions, etc). Nevertheless, as with any method, each of those discussed in this review has inherent pros and cons. Moreover, there will always be the trade-off between spatial and temporal resolution to consider for any imaging experiment. Having provided an overview of numerous super-resolution microscopy techniques and their most suitable applications, we have aimed to provide a framework for selection of the most appropriate method depending on the experimental question, specimen of interest, and available instrumentation.

## Conflict of interest

The authors declare that they have no conflicts of interest with the contents of this article.
